# “Going Beyond Darkness”—Lingering Images and Ideation of Self‐Destruction

**DOI:** 10.1111/jpm.13137

**Published:** 2024-11-15

**Authors:** Nina Veetnisha Gunnarsson

**Affiliations:** ^1^ Department of Social Work, School of Health and Welfare Jönköping University Jönköping Sweden

## Abstract

**Purpose:**

This paper is a personal reflection on the persistence of self‐destructive thoughts and ideations in individuals with a history of self‐destructiveness.

**Background:**

Personal narratives are essential for understanding mental health and illness, particularly for those with a history of self‐destructive behaviour. I, a researcher with lived experience of mental illness and self‐destructiveness, highlight the importance of presenting them in their raw and unmediated form.

**Methods:**

This paper is a first‐person narrative where I use my own personal experiences to illustrate and analyse the persistence of self‐destructive thoughts.

**Summary**

- What is known on the subject?
  ○ Individuals with mental health issues or trauma may experience a persistent longing or ideation towards self‐destruction.
  ○ These thoughts can bring a paradoxical sense of peace and comfort.
- What this paper adds to existing knowledge?
  ○ This reflection offers a personal insight into the ongoing allure of self‐destructive thoughts for someone who has experienced and moved beyond active self‐harm.
  ○ The first‐person narrative highlights the complexity of these thoughts and their role in providing a sense of relief from monotony and internal conflict.
- What are the implications for practice?
  ○ Mental health practitioners should recognise that self‐destructive thoughts can persist even in individuals who seem to have recovered.
  ○ These thoughts can be vivid and intrusive, potentially impacting the individual's well‐being.
  ○ Mental health nursing must include space for patients to share these unmediated experiences, helping practitioners better understand and support their ongoing struggles.

## 1 Introduction

This paper is an exploration of the complex relationship between self‐destructive behaviours and the search for identity and meaning. Here, I reflect on how self‐destruction, often glamorised in music and autobiographies, can provide a sense of control, identity, and relief from emotional pain. I delve into the paradox that self‐destructive thoughts and behaviours, while harmful, can also serve as coping mechanisms and contribute to a sense of aliveness and self‐cohesion. I also question societal narratives about self‐worth and the pressures to conform to ideals of success and self‐fulfilment. There is also a need for a deeper understanding of these behaviours in mental health care, suggesting that even those who appear recovered may still grapple with intrusive thoughts and fantasies of self‐destruction. First‐person narratives may include autobiographies, autoethnography, and other kinds of personal reflections (Adams, Holman, and Ellis 2022). First‐person narratives offer unique insights into personal experiences to provide a deep, insider perspective and can be powerful tools for illustrating lived experiences in fields like mental health, where understanding the subjective experiences of individuals can inform empathetic care practices and contribute to the formation and growth of storytelling communities (see Crossley and Crossley 2001). This personal narrative urges mental health practitioners to recognise the complex role of self‐destructive ideations, which may offer paradoxical comfort or identity even after recovery. As a scholar with lived experiences of a self‐destructive past, I want to use my platforms to speak about how mental and emotional challenges are sometimes never fully resolved.

## 2 To Go Beyond Darkness

My second first name, Veetnisha, is an Indian (meditation) name that means “to go beyond darkness.” This personal reflection focuses on that meaning—having gone beyond darkness but still imagining and daydreaming about it, the self‐destructiveness. In this reflection, I draw on memories of past self‐destructive behaviours and the persistent ideations that I manage on a regular basis. I begin here with a prologue of thoughts and reflections that flash through my mind as I attempt to make sense of these experiences.

While writing this, I sit in my armchair and listen to music. It's this music that sets my fantasies in motion—or is it more than that? My desires, my expectations, my deepest reality. I don't know. “We must live, we must keep on living,” I hear the singer sing. Yes, maybe that's true—and yet it isn't. Why must we? Why must I?

“Dry my tears, it's so damn sad for me. Poor wretch.” Another song by the same artist. Maybe not even relevant to this text, but still what surfaces within me, in my thoughts, makes me wonder: is this something I've started that I should really write about? All these lyrics are about love, for another, for a partner, a lover. But that's not what I perceive or what stirs within me—love for a partner. I left that behind some time ago.

What I perceive, and feel is the sense of ruin. Self‐ruin. Maybe I don't want to die at all but just cause myself ruin. I want to destroy myself, my soul, my body. I want to bleed, maybe bleed out, want to see myself in a pool of blood, and I want to see my skin open up and the blood gush out. I want to drink myself senseless; I want to stop thinking and stop being bored and restless. I just want to be in the moment, in a now that demands nothing more from me than to lie on the floor. Bloody and senseless. Torn apart and destroyed.

I do not (any longer) suffer from mental illness or a psychiatric disorder per se. Sure, they say I have ADHD (and I do medicate for it and need the medications too) and I have been given various diagnoses throughout my life, but today I see myself as mentally healthy, well, and as normal as the neighbour next door. Yet, self‐destruction is in my thoughts sometimes, perhaps far too often and far too vividly and literally. I have already had times in my life when I was there, where my fantasies take me. I have lain drunk and bloody on the floor. I have destroyed myself and my body countless times throughout my life. However, that is, not where I am today. Still, it is these thoughts that give me peace and make me feel free. What does that really mean? Why is it that I go there, to this dimension, when I think of something that lifts my spirits, makes me less restless and bored? What is it about self‐destruction that brings me peace?

Do individuals who have experienced trauma or engaged in self‐destructive behaviours for various reasons share my perception? That life can feel stagnant, and self‐destruction becomes the sole source of vitality, injecting a new energy absent in the monotony of everyday life? Or is this perception a result of inherent psychological conditioning? From cradle to grave. The answer eludes me.

It is perhaps inadvisable to write about this, particularly with the intent of publishing these reflections. As a middle‐aged lecturer and researcher with grandchildren, I am acutely aware of how such disclosures might be perceived. How should one approach the experiences of a seemingly successful woman who spends her evenings romanticising and longing for self‐harm? Nevertheless, if I do not address this topic, who will? I refuse to believe that I am alone in having these thoughts and feelings. To suggest otherwise would be an interpersonal impossibility.

## 3 Images and Thoughts About Self‐Destruction

I have had my demons, and perhaps, I am fooling myself into believing that I no longer see them, feel them, or interact with them daily. But no, I am no longer tormented by demons, darkness, and despair. I wake up early every morning, eager for a new day. I am active and productive and social even though I don't take care of my body. I don't exercise, eat too often and unhealthily, and I certainly drink a lot. I use nicotine ‘snus’ (Swedish) but no longer smoke. I drink soda and eat crisps whenever I want. I could take care of myself and the body I have more, absolutely. But I enjoy life.

I don't live in misery (anymore). I work full‐time, I'm a university lecturer and researcher, productive, always sober, straightforward, and “well‐groomed” every day at work. I'm efficient in my work and well‐liked by my colleagues. I have friends, a close family, and people who care about me and whom I care about. I don't live in misery in any way, quite the opposite. Yet, I have this longing, this fantasy, this alternate reality that I don't consciously hide, that I don't exactly conceal, but that I also don't fully openly acknowledge.

I have read numerous autobiographical books about people who have had tough lives, who have harmed themselves in various ways, engaged in self‐destructive behaviours, lived with various addictions, eating disorders, mental health issues, suicide attempts, traumas, and other experiences that can be described as living with demons and darkness. Not all of these stories are about a conscious self‐destructive lifestyle or a longing for it. However, it is common to remain in what is, after all, a form of security—something familiar, something one is accustomed to—compared to a life one knows nothing about.

A study from Iceland involving men who had experienced sexual violence showed that they often thought of suicide as a means of escaping from themselves and their inner suffering (Tryggvadottir, Sigurdardottir, and Halldorsdottir 2019). Suicidal thoughts and self‐destructive behaviour provided a way to find peace and alleviate significant emotional pain. However, these men's self‐destructive tendencies were often acted upon, although suicidal thoughts could persist in the form of images and ideations and offer emotional relief.

In another way, one might not believe one is worthy of any other life. That is the take‐home‐message of plenty autobiographies on the topic of mental illness, addiction, and self‐destruction. There is a saying I once heard in a treatment center, coming from Nelson Mandela's inaugural speech 1994 and the first three lines go:

“What we fear most is not that we are inadequate. What we fear most is that we are powerful beyond measure. It is our light, not our darkness, that most frightens us.

There are certain “grand narratives” that we are raised with and learn to believe in and proclaim to ourselves and others. One of these is the idea that you shouldn't think too highly of yourself (at least in my country, this is a common mantra‐ in Swedish “jantelagen”). Another narrative is about taking care of yourself (and your body) and fulfilling your own potential (the cradle of individualism in the neoliberal modern world). Yet another narrative is that you are the architect of your own fortune (so if you don't succeed in life, it is essentially your own fault).

In these three narratives, there are both similarities and contradictions. The first one is in direct opposition to the last two. The latter two are at the core of the modern world and the self‐reflective individual, emphasising the idea that you are your own project, which you should nurture, work on, and discipline. Giddens (1991), Beck (1992), and Foucault (2017) all described these aspects of the (post)modern individual in industrialised countries. If you fail to take care of yourself, fulfil yourself, and discipline your body and mind, who are you then and what are you worth?

Just as Mandela said, it is our light and not our darkness that we fear. Perhaps, it is precisely the light within us—the good, the beautiful, the remarkable, the unique, the generous—that we are afraid of. And why do we fear it? I believe it is because we have been deceived into thinking that the project of ourselves is somehow sufficient for us to feel whole and valuable. The project itself is impossible to fulfil for everyone, as we are valued differently in the eyes of society and one another. If we fail according to social and cultural standards of what constitutes a successful, self‐fulfilling individual, it is us that are at fault—not the standards, ideals, or ideology behind them. The fault lies with us, the individual, with you, with me, and not with the narrative.

Manning (2020) argues that self‐destruction, such as suicide, can be a way to handle or respond to conflict, serving as a form of conflict management or social control. It can manifest as escape, protest, or punishment. For instance, if self‐destruction is a way to escape, who is the person trying to escape from—an external enemy? Who am I trying to escape from by fantasising about and longing for the self‐destructive part of me? Is it an external enemy or an internal escape (from myself)? Or is it a protest against my own individual circumstances (which, though not unhappy, do involve hardships) or a protest against the self‐fulfilling, self‐reflexive self‐project of the modern individual, to which I, like most of us, am subjected?

I believe what I am escaping from is the daily routine, the monotony of everyday life that demands nothing from me and doesn't trigger any adrenaline. I think I probably should have had a job that brought excitement to my daily life, gave me a greater sense of purpose, and demanded more from me than academia does. Academia is not for adrenaline junkies, and I suppose that's what I miss and what I get from thinking about, wishing for, and fantasising about a self‐destructive lifestyle. I escape boredom in my thoughts. Perhaps it is not more dramatic or complicated than that. It is rather banal and, I believe, not particularly unique. Perhaps my past suicidality, self‐cutting, self‐burning, and drug/alcohol use were signs of the same story. It is just that now I am a middle‐aged researcher and grandmother—a very present grandmother and, for my grandchildren, a precious one. But why do suicidal thoughts, ideation, and self‐destructiveness allure me and make me feel excited and exhilarated? Over periods of my life, my past self‐destructiveness was a misery, although it did at times save my life. When all I wanted was to die, the self‐injury kept me alive. But why would I want misery to (re) enter my thoughts, even though they are today only thoughts?

In psychological theories, so called self‐theories, the idea of self‐destruction is a motivated action that, according to Stolorow (1975) means that self‐inflicted pain can paradoxically serve as a means of self‐enhancement. Engaging in ‘masochism’ and self‐destructive behaviours might actually elevate self‐esteem and self‐worth. These actions can create a sense of continuity over time and help maintain a cohesive self‐identity by establishing clear boundaries between the self and the external world. Physical pain on the body's surface can aid a fragmented self in re‐establishing bodily boundaries (self‐differentiation) and inner cohesion, while also fostering a sense of aliveness, albeit through painful experiences.

Even though it is an old idea, it still resonates with me and my experiences. So, do I have a fragmented self, and as such, need to reaffirm my bodily boundaries and a sense of inner consistency? I don't know. But I do know that simply thinking about harming myself in different ways fosters a sense of aliveness in me.

Another researcher, Sacksteder (1989), suggested that self‐destruction is a form of negative identity. He claims that having a negative identity is better than having no identity at all. There have been periods in my life when this perception has undoubtedly resonated with my experiences. The inclination towards self‐destructive behaviour functioned as a form of identity, something uniquely my own, which I could regulate and utilise to render myself ostensibly more interesting than I otherwise perceived. While such behaviour might have appeared negative from an external standpoint, through the lens of others, it did not bear the same negative connotation internally, within my personal sense of self. Because who does not want to be unique in some way?

The allure of self‐destruction for me seems to lie in its ability to break the monotony of daily life and provide a sense of identity and aliveness. Despite leading a fulfilling life, the temptation of past behaviours remains with me, highlighting the complex relationship between self‐destruction, identity, and societal expectations.

## 4 Conclusions

What relevance does my personal narrative have to mental health nursing? Personal narratives serve as powerful instruments for illustrating the lived experience of mental illness and its implications. As both a researcher and an individual with a history marked by mental illness, addiction, and self‐destructive behaviours, I possess a distinctive insider perspective that I find indispensable in my scholarly work. Previously, I have explored the shame that emerges in intimate relationships and the role of self‐harm as a coping mechanism for that shame (Gunnarsson 2021). Additionally, I have authored a personal reflection on the significance of self‐harm scars (Gunnarsson 2022) and have examined my own experiences with self‐harm during middle age, addressing the stigmatisation it brings, and the perceptions held by others, including healthcare professionals, who often view individuals like me as childish, immature, and manipulative (Gunnarsson 2023).

I have worked with autoethnographic (like) approaches because my first‐person narrative is relevant to the stories of others with similar experiences and to the broader cultural narratives surrounding mental health and illness. First person narratives are based on the idea that the personal and the social are mutually inclusive (Church 1995). The personal is social, and the social is personal.

In researching self‐harm and self‐destructive behaviours, it is nearly impossible to exclude my own experiences. It would require me to disregard the knowledge and insights I have personally acquired. Consequently, I frequently employ my own experiences to understand and explain these phenomena. While some scholars may perceive this approach as narcissistic and self‐indulgent, I, along with other researchers, contend that it is not feasible to separate our own cognitive and emotional lives from those of others (Adams, Holman, and Ellis 2022). We share common experiences and emotions that enhance our mutual understanding. This interconnectedness allows us to recognise aspects of ourselves in the experiences and life trajectories of others.

Also, I believe that understanding these experiences from both a personal and social perspective can be beneficial for healthcare and psychiatry, not ‘sugarcoating’ experiences of self‐destructiveness but presenting them in a raw, unprocessed, and moreover, unmediated form (not filtered through someone else, such as via interviews) is valuable for the science of mental health and the knowledge that mental health nurses needs to engage with every day, that is, understanding patients in mental health care.

Although this lived experience article is not about self‐destruction per se (I have written another piece on that), it explores the lingering image and thoughts of self‐destructive behaviours. I believe there is still much to be learned about the persistence of self‐destructive thoughts and ideations in individuals who have been highly self‐destructive over long periods of their lives. The desire, longing, or escape—whatever one chooses to call it—may linger with a person their entire life. This is significant for mental health nursing practice because it may mean that even if someone appears to have ‘recovered’ from a mental health issue, they may still be immersed in images of darkness and despair. Images that are intrusive and vivid can be just as real and can be detrimental to the person. I suggest that practicing mental health nurses and other practitioners need to follow up on individuals' self‐destructive ideations and thoughts, even when treatment or the natural course of mental health issues have seemingly been effective.

## Conflicts of Interest

The author declares no conflicts of interest.

## Data Availability

Data sharing is not applicable to this article as no new data were created or analyzed in this study.
